# Supplementary material for: Mechanisms governing the pioneering and redistribution capabilities of the non-classical pioneer PU.1
Source: Nat Commun. 2020 Jan 21;11:402. doi: 10.1038/s41467-019-13960-2 (PMC6972792; doi:10.1038/s41467-019-13960-2)
Supplement: Supplementary file 7 — Source data [file 41467_2019_13960_MOESM7_ESM.zip › Source_Data/Figure6/Figure6B_MotifScanOutput/ETS1_PU.1/homerResults/motif42.similar.html]

motif42

## Information for motif42

T
G
C
A
C
T
G
A
T
C
A
G
C
T
G
A
A
C
G
T
A
C
T
G
A
C
T
G
A
G
T
C
A
C
T
G
T
C
A
G
G
A
T
C
A
T
G
C
A
T
G
C
A
T
G
C
  
Reverse Opposite:  

T
A
C
G
T
A
C
G
T
A
C
G
C
T
A
G
A
G
T
C
T
G
A
C
C
T
A
G
A
G
T
C
A
G
T
C
G
T
C
A
A
G
C
T
A
G
T
C
A
G
C
T
A
C
G
T
  

|  |  |
| --- | --- |
| p-value: | 1e-29 |
| log p-value: | -6.830e+01 |
| Information Content per bp: | 1.740 |
| Number of Target Sequences with motif | 196.0 |
| Percentage of Target Sequences with motif | 1.24% |
| Number of Background Sequences with motif | 104.1 |
| Percentage of Background Sequences with motif | 0.32% |
| Average Position of motif in Targets | 96.7 +/- 55.6bp |
| Average Position of motif in Background | 111.7 +/- 60.2bp |
| Strand Bias (log2 ratio + to - strand density) | -0.1 |
| Multiplicity (# of sites on avg that occur together) | 1.06 |
| Motif File: | file (matrix) reverse opposite |

### Similar de novo motifs found

|  |  |  |  |  |  |  |  |
| --- | --- | --- | --- | --- | --- | --- | --- |
| Rank | Match Score | Redundant Motif | P-value | log P-value | % of Targets | % of Background | Motif file |
| 1 | 0.943 | A T C G A C T G A G T C A T G C A C T G A G T C A G T C C G T A A C G T A G T C A C G T A C G T A C T G | 1e-27 | -62.333181 | 0.99% | 0.23% | motif file (matrix) |
| 2 | 0.928 | C A T G T A G C T G A C A T C G T A G C T G A C G T C A C A G T A C T G G A C T G A C T | 1e-26 | -61.904172 | 2.39% | 1.07% | motif file (matrix) |
| 3 | 0.948 | C T G A T C G A C A T G T G C A A G C T A T C G A T C G A G T C C A T G T A C G T A G C A G T C | 1e-21 | -49.100605 | 0.93% | 0.26% | motif file (matrix) |
